# Supplementary material for: Open banking: A bibliometric analysis-driven definition
Source: PLoS One. 2022 Oct 3;17(10):e0275496. doi: 10.1371/journal.pone.0275496 (PMC9529117; doi:10.1371/journal.pone.0275496)
Supplement: S1 Annex — (DOCX) [file pone.0275496.s001.docx]

# S1 Annex. Open banking definitions.

| **#** | **Definition** | **Author** |
| --- | --- | --- |
| 1 | [...] in order to promote and facilitate data sharing interactions between banks and third-party service providers, the eight major British banks were mandated to develop jointly a single, open, standardised application programming interface (API) freely available for the whole industry. | [49] |
| 2 | This technology focuses on improving customer experience in more than one way. Use of application programming interfaces (APIs) is imperative to define how financial data can be created, shared, and accessed securely and efficiently. This technology forces the banks to be competitive with their counterparts. Hence, they are compelled to bring down the cost and implement improved technology for excellent customer-care experience. This technology requires banks to be transparent with their online and internal banking information, which must be unbiased and accurate. Additionally, it helps lenders to analyze their borrowers’ financial situation and the risks related to it. | [50] |
| 3 | European Union (EU) has forced banks to open up and make their customer information available with their permission so that other players in the financial market (TPP) can use them and take some of that work. | [51] |
| 4 | [...] regulation which mandated that banks disclose data that they hold on individual consumer 0transactions—as well as on their own prices and services—to third-party services providers, such as price-comparison tools. | [2] |
| 5 | [...] regulations that would require banks to securely share customer data with authorized third parties. These […] regulations are designed to increase consumer choice and mobility, innovation, and competition in the financial services industry. | [52] |
| 6 | [...] requiring banks to release and make available through an open-APIs [...] a wide range of reference and product information, including the prices, charges, terms and conditions for all personal and business current account products (including overdrafts) and small business lending products and service quality indicators (for example customer recommendation scores) specified by the CMA in its remedy on service quality and at the time required by this remedy. | [53] |
| 7 | [...] require the nine largest banks to provide open standardised API data on their retail customers and SME account data to third parties (where the consumer elected to do so). This uses digital technology to create the possibility of a new market for firms to design applications that advise and help consumers to better manage their money and the financial products they use. | [54] |
| 8 | [...] with technology firms. It does so by granting a right to the user of payment services to make use of payment initiation and account information services, even where the payment institutions have not entered into a contract with the respective (new) service provider. It assigns to clients an ownership right over their data and provides at the same time a specific use case for the data subject’s data portability right granted by Article 20 of GDPR, thereby linking the PSD 2 initiative to the GDPR objective laid out above. Open banking is the regulatory response to the anti-competitive tendencies of the data economy where the size of the data pool determines competitive strength92 and where technology firms like Amazon, Google and others have foregone profits for years to build dominant platforms. | [16] |
| 9 | Open banking is based on the bank’s ability to engage non-banking intermediaries ecosystems and third parties (eg FinTech companies) to the banking service provision and to interact with them through such technologies as application programming interfaces (API). | [13] |
| 10 | [...] require inter alia banks and certain e-money institutions (“EMIs”) to publish application programming interfaces (“APIs”). These APIs mainly enable licensed payment initiations (“PISP”) and account information service providers (“AISP”) as well as banks, to either initiate payments or retrieve account information after having obtained the end-client’s consent. | [55] |
| 11 | The exchange of consumer data between banks and other FSPs (i.e., data holders), on the basis of customer consent, with other FSPs and/or TPPs such as fintechs (i.e., AISPs and PISPs—both known as data users). Although payment initiation is an important element of open banking from a financial inclusion perspective (as discussed in Section 3), it is not essential to the functioning of open banking, and therefore it is not included in its definition. | [56] |
| 12 | [...] allows consumers to opt in to sharing their financial transaction data with fintechs and allows fintechs to use these data to develop products and services customized to individual needs. | [57] |
| 13 | [...] a new kind of business ecosystem characterized by the widespread use of data-enabled services to deliver innovative and more competitive services to consumers. Such development builds on the use of open application programming interfaces (APIs) that enable consumers to take advantage from their account data by sharing them with authorized third parties. | [58] |

Table 3: Definitions of Open banking (1/3)

| 14 | [...] a new regulatory and technological framework called “open banking” raises the possibility of consumers being able to task trusted intermediaries with automatically analyzing their financial data, nudging them to achieve their goals, and switching them to better products, all in order to reduce the substantial inefficiencies in their financial lives. | [59] |
| --- | --- | --- |
| 15 | […] initiative that] allows customers to share their data securely with other banks and third parties (e.g., challenger banks, fintechs, utility companies, and other businesses) to provide a seamless route to accessing new products and services. | [60] |
| 16 | […] remedy […] which enables customers to share transaction and other data with regulated third parties through secure APIs, thereby enabling third parties to offer services like budgeting advice or comparisons of different products based on customer needs. | [61] |
| 17 | [...] enables its customers to easily and securely share their banking data with trusted groups. | [62] |
| 18 | [...] a universal undertaking that endorses a customer’s right to share financial information with third parties. | [63] |
| 19 | [...] an initiative which facilitates the secure sharing of account data with licensed third parties through Application Programming Interfaces (APIs), empowering customers with ownership of their own data. The initiative aims to increase competition in retail banking by developing innovative products and services which will bring increased value to customers. | [15] |
| 20 | [...] framework to give customers access to and control over their financial data. As the experiences of the United Kingdom and Australia have shown, an open banking framework allows consumers to direct banks to securely share only the financial data that they choose, for the duration that they choose, through the use of application programming interfaces, or APIs. | [64] |
| 21 | Open Banking is a framework designed to give customers a right to direct that the information they already share with their bank be safely shared with others they trust. It is intended to give customers more control over their information, leading to more choice in their banking and more convenience in managing their money, and resulting in more confidence in the use of an asset mostly undiscovered by those customers – their data. | [19] |
| 22 | Customer data [...] have become more “open” to external third parties, whenever customers who generate these data consent to share them. Open banking, an initiative led by several governments, including Australia and the United Kingdom, leads such a shift toward the open-data economy. | [31] |
| 23 | [...] generic term for regulations that oblige financial institutions to provide secure channels for customers to share their financial data with third parties, and in some cases requires the financial institution to provide third parties with the ability to move customer funds. | [65] |
| 24 | [...] a platform cooperation mode, which uses open application programming interface (API) technology to realize data sharing between financial institutions and third-party providers, so as to improve customer experience. | [66] |
| 25 | [...] is a sharing between incumbent financial institutions and third party financial service providers. | [67] |
| 26 | [...] requirements whereby incumbent financial intermediaries must share client data with third parties, including potentially innovative new competitors. | [17] |
| 27 | The Open Banking is the process which brings together the Financial service resources and Open Data resources and enables a path for Financial Services Providers to develop new methodologies of delivering Financial Services to a customer. Open Banking is based on the use of API access to the data pools, to the infrastructure of the regulatory compliant and to other financial services resources. If a bank entity will join the Open Banking Model it will consume and provide APIs also it will be a new Financial Service Provider. | [68] |
| 28 | [...] an open banking regime will make it easier for individuals, including small businesses, to share their own transaction data securely with third-party service providers, such as potential lenders. | [69] |
| 29 | [...] enables personal customers and small businesses to share their data securely with other banks and with third parties, allowing them to compare products on the basis of their own requirements and to manage their accounts without having to use their bank | [9] |
| 30 | […] involves third parties (e.g., fintech) accessing a customer’s account using customers’ personal security credentials, and in some cases, initiating transactions on their behalf. | [48] |
| 31 | [...] standardized interfaces to financial institutions’ data. These interfaces enable third parties, in particular FinTech companies, to access users’ bank account information and initiate payments through well-defined APIs. All around the world, API banking is being promoted by law or by industry demand: In Europe, the Payment Services Directive 2 (PSD2) regulation mandates all banks to introduce Open Banking APIs by September 2019 [2]. The US Department of the Treasury recommends the implementation of such APIs as well. In South Korea, India, Australia, and Japan, open banking is being pushed by large financial corporations. | [70] |

Table 4: Definitions of Open banking (2/3)

| 32 | [...] legislation [that] allows European financial services to become truly platformized by permitting third parties (usually tech firms) to access mountains of customer data sequestered inside banks, insurers, and other financial services providers. [...] permits technology companies to access banks’ customer data with customers’ permission. The digital key to unlocking transparency in platformized markets is the open API (application programming interface), a software tool which allows data sharing between websites and online services, making it possible for tech companies to have a transparent view of financial providers’ customers. | [71] |
| --- | --- | --- |
| 33 | [...] allow third-party access to consumers’ bank data (with the consumers’ consent) and are becoming a fundamental tool of digital disruption. | [72] |
| 34 | [...] a financial services term as part of financial technology that refers to: The use of open APIs that enable third-party developers to build applications and services around the financial institution; Greater financial transparency options for accountholders ranging from open data to private data ; The use of open-source technology to achieve the above. | [73] |
| 35 | [...] the use of open application programming interfaces (APIs) that enable third-party developers to build applications and services around the financial institution;, greater financial transparency options for account holders ranging from open data to private data, and the use of open-source technology to achieve the above; can be naturally evolved into a new ecosystem of data marketplaces where participants can buy and sell data. | [24] |
| 36 | Open banking describes a special kind of financial ecosystem. The ecosystem provides third-party financial service providers open access to consumer banking, transaction, and other financial data from banks and nonbank financial institutions through the use of application programming interfaces (APIs). | [32] |
| 37 | [...] policie [that] require banks to provide access to their customers’ payment account data to third-party providers of payment services, subject to customer consent, to enable them to offer new, differentiated services based on the use of these data. | [74] |
| 38 | [...] involves opening up banking systems (functionality and customer data) to third parties to allow them to provide services directly to customers. | [75] |
| 39 | [...] a technological platform that supports the use of third-party digital shopping assistants (labelled aggregators), through the adoption of standard application programme interfaces (APIs), with the aim of reversing the persistent low level of switching activity due to consumer disengagement. | [76] |
| 40 | [...] all kinds of things, from a remedy to an ecosystem, or most often: a (business) model of some sort. Its purposes are considered to be providing new (‘better’, ‘customer-centric’) services to customers and improving competition in the banking market by letting ‘third parties’ in. | [14] |
| 41 | [...] will enable banks to share their data with third-party service providers. It will allow customers to open new accounts or make their banking transactions easily from a single user interface that can be fed by data by many banks and fintech companies. In this way, consumers will have the chance to compare the offerings of various banks and make their transactions easily since the information about customers’ accounts will be aggregated and viewed by the customers in one interface. | [77] |
| 42 | [...] a standardised framework for sharing bank customer data. Open banking reduces barriers to entry and eliminates banks’ monopoly over their customers’ data, making it easier for FinTech firms to innovate. The secure sharing of customer banking data has the potential to promote financial system soundness. By increasing competition and unbundling banking services across a larger range of firms, it reduces the systemic importance of large banks. Greater sharing of customer data may also create opportunities for more personalised financial products and lower switching costs (which may promote market discipline). Open banking also presents risks, including making the banking sector more prone to cyber risk. | [78] |
| 43 | [...] a model in which banking data is shared through Application Programming Interface (API) to third parties. | [79] |
| 44 | [...] is an emerging financial services model that focuses on the portability and open availability of customer data held by financial institutions. | [80] |
| 45 | [...] the EU’s regulatory response to the anti-competitive tendencies of the data economy where the size of the data pool determines competitive strength and where technology firms like Apple, Amazon, Google and others have foregone profits for years to build dominant platforms. | [5] |
| 46 | Evolution of banking, leading to more transparency, customer choice and customer control over personal data. | [81] |
| 47 | [...] involves sharing business services such as data, algorithms and transactions with business ecosystems of employees, customers, partners, fintechs and others. Open banking enables business ecosystems to build new apps, products, and services; match buyers and sellers; and create new business models. | [26] |

Table 5: Definitions of Open banking (3/3)
