# Supplementary material for: Open banking: A bibliometric analysis-driven definition
Source: PLoS One. 2022 Oct 3;17(10):e0275496. doi: 10.1371/journal.pone.0275496 (PMC9529117; doi:10.1371/journal.pone.0275496)
Supplement: S2 Annex — (DOCX) [file pone.0275496.s002.docx]

# S2 Annex. Analytical approach.

Mathematically, different options exist to perform clustering analysis. The most popular approach is MDS [82]. MDS is an analysis technique that aims at locating similarity or relatedness between any two items based on the distance between them, rooted in the premise that the smaller the distance, the stronger the relationship. In this study, however, we apply VoS, a version of MDS that properly factorizes association strength when ordinal or interval factors are applied, as in the case of co-word analysis [83]

VoS provides a low-dimensional visualization where items are positioned so that the distance between any pair reflects their similarity as precisely as possible. Thus, VoS minimizes the weighted sum of the squared Euclidean distances between all pairs of items, assuming that the higher the similarity between two objects, the higher the weight of their squared distance in the summation [84]. The objective function to be minimized in VOS is given by:

$E(\mathbf{X}; \mathbf{S}) =\sum_{i<j} {s_{i,j}\left\| x_{i-}x_{j} \right\|}^{2},$ [1]

where $\left\| .. \right\|$ denotes the Euclidean distance, $s_{i,j}$ denotes the similarity between object *i* and object *j*, and the vector xi = ($x_{i,1}$, . . . , $x_{i,m}$)$\in\mathbb{R}^{m}$denotes the *i*-th row of ***X*** and contains the coordinates of object *i*.

The minimization of the objective function is subject to:

$\sum_{i<j} \left\| x_{i}-x_{j} \right\|=1$ [2]

For visualization purposes, each object is positioned close to its ideal coordinates, given by:

$c_{i}\left( \boldsymbol{X};\boldsymbol{S} \right)=\frac{\sum_{j} s_{i,j}x_{j}}{\sum_{j} s_{i,j}}$ [3]
