# Supplementary material for: Open banking: A bibliometric analysis-driven definition
Source: PLoS One. 2022 Oct 3;17(10):e0275496. doi: 10.1371/journal.pone.0275496 (PMC9529117; doi:10.1371/journal.pone.0275496)
Supplement: Attachment — Submitted filename: PONE-D-22-03690 Review report.docx [file pone.0275496.s007.docx]

**Review of PONE-D-22-03690**

**“Open banking: A bibliometric analysis-driven definition”**

**Summary and Strengths**

As a relatively new concept, open banking has generally been considered as the transformational lever of the retail banking industry. However, its theoretical conceptualization was underdeveloped in the literature. This study presents a potential generalized definition of open banking based on bibliometric and discourse analysis. This paper is interesting and provides timely insights into the literature. Below I provide comments/suggestions that can hopefully help improve the paper.

**Comment 1:**

This paper is not very well organized and may confuse your audience. For instance, the authors mention that the existing literature can be grouped into four topics in the Introduction section. In addition, the Literature Review section also includes a review of prior literature under four different perspectives. So, I thought the prior literature groups these topics/perspectives. But when I read the Results section, it seems that these four topics are grouped based on your clustering analysis. Authors should clarify how you group prior literature into those topics. If the prior literature suggests it, what are the differences between this and the clustering results? If those are the output of the clustering analysis (your findings), they should be discussed in the Results section instead of the literature review.

**Comment 2:**

The motivation of the study is underdeveloped. On page 6, there is an initial definition of open banking. So, what is the motivation behind this study to propose a generalized definition? Does the existing one have any limitations? The authors also discuss the lack of evidence about the benefit to end customers. But I don’t think this study answers this question by proposing a definition. So, I would recommend authors re-organize the literature review section and highlight the relevant motivations of this study.

**Comment 3:**

The sample period of the 282 documents is from 2003 to 2021. However, the concept of “open banking” was first introduced in 2015; it is possible that the term “open banking” referred to something different in the early studies? Authors may want to include an explanation to eliminate some concerns about the sample validity.

**Comment 4:**

48 definitions were extracted during the bibliometric analysis. However, it is not clear how these definitions were identified and extracted. Does the authors manual read all the abstracts or apply any algorithms? Were similar definitions extracted from different papers combined? As less than 20% of the papers have a definition, is that possible that most papers provide a definition in the main body instead of abstract?

**Comment 5：**

I would recommend authors provide more details about the clustering approach and interpretation of each cluster. The main idea is to ensure validity. Do multiple authors code them independently and achieve an agreement? It seems to me that the interpretation is kind of arbitrary. For instance, the last cluster is coded as regulation. But except for the keyword “regulation”, other keywords like “payment”, “transaction”, “market” are somehow irrelevant to the topic of regulation.

**Comment 6:**

In the analysis of definition, this paper decomposes 48 identified definitions into 8 elements. How do those 8 elements identified? Are they developed by authors or based on literature? Authors may need to provide additional explanations.

**Comment 7:**

The findings of the paper make potential contributions to the literature. However, authors did not systematically discuss their contributions to the literature. I would recommend including a paragraph of contribution in the Introduction section.

**Minor comment:**

The format of some citations needs to be adjusted. For instance, on page 3, change “To date, … shaping forces of the future (Ziegler, 2021) (Brackert et al., 2019).” to “To date, … shaping forces of the future (Brackert et al., 2019; Ziegler, 2021).”

Many subsections are numbered incorrectly. For example, on page 12, change “2.1.2.” to “3.1.2.”.

On page 13, change “5.000” to “5,000”.

On page 15, change “10.000” to “10,000”.
