# Supplementary material for: Open banking: A bibliometric analysis-driven definition
Source: PLoS One. 2022 Oct 3;17(10):e0275496. doi: 10.1371/journal.pone.0275496 (PMC9529117; doi:10.1371/journal.pone.0275496)
Supplement: Attachment — Submitted filename: Response to Reviewers.docx [file pone.0275496.s008.docx]

May 22, 2022

Tawei Wang

Academic Editor

*PLOS ONE*

Dear Editor:

First, we would like to thank the referees for all the comments, suggestions, and ideas they have given us to improve our work. This paper is considerably more transparent after implementing recommended changes, and its motivation and contribution to the literature are significantly more robust.

To provide an answer to and solve the concerns raised, in this Rebuttal Letter, we use the following structure:

1. We answer the Journal Requirements regarding formatting, including all the changes needed (e.g., citation method).
2. We show our approach to the significant concerns signaled by the two Reviewers as a common answer.
3. Then we make a step-by-step personalized response to every comment raised by each reviewer, using a Table to pull out the comment and in a separate column detailing our response (comments in black, response in blue, additions/changes to the article in green).

We hope that our answers, modifications, and actions in the revised version of the manuscript and Rebuttal Letter have addressed the raised concerns satisfactorily.

Again, thank you so much for your suggestions, comments, and wishes.

Yours sincerely,

Gorka Briones de Araluze

DBA Candidate

Universidad Pontificia Comillas.
Alberto Aguilera, 23, 28015, Madrid, Spain

[gbrionesde@comillas.edu](mailto:gbrionesde@comillas.edu)

(Corresponding author)

**1. Journal Requirements**

**1.1. When submitting your revision, we need you to address these additional requirements. Please ensure that your manuscript meets PLOS ONE's style requirements, including those for file naming. The PLOS ONE style templates can be found at** [**https://journals.plos.org/plosone/s/file?id=wjVg/PLOSOne_formatting_sample_main_body.pdf**](https://journals.plos.org/plosone/s/file?id=wjVg/PLOSOne_formatting_sample_main_body.pdf) **and**

[**https://journals.plos.org/plosone/s/file?id=ba62/PLOSOne_formatting_sample_title_authors_affiliations.pdf**](https://journals.plos.org/plosone/s/file?id=ba62/PLOSOne_formatting_sample_title_authors_affiliations.pdf)

All the style requirements have been implemented in the revised version of the document to ensure the manuscript meets PLOS ONE´s style requirements.**1.2. We note you have included a table to which you do not refer in the text of your manuscript. Please ensure that you refer to Table 2, 3, 4, 5, and 6 in your text; if accepted, production will need this reference to link the reader to the Table.**

Tables 2, 3, 4, 5 and 6 are now referenced in the text.

**2. Comments regarding the major concerns exposed by the reviewers**

From a careful reading of all the comments, we have understood four major suggestions to improve the paper: issues with structure, lack of motivation, lack of clarity regarding the methodological approach and problems with the discussion.

**2.1. Structure**

Both reviewers point out the need for a revised structure for the article. After careful re-reading, we agree that the current one can be confusing for two main reasons: a lack of focus in the literature review and the concurrence of two methodological approaches.

Firstly, the Introduction and Literature review sections are confusing, as both contain information that might be redundant for the article's goal. Thus, we have restructured these sections and left in the Literature review section only the relevant information to understand the gap in the academic literature this article tries to cover.

Then, the concurrent usage of two methodological approaches, a bibliometric approach and a discourse analysis approach, might generate some confusion. To solve this, we have written an introduction to the methodology section to explain further why both methods are required. First, a bibliometric and clustering analysis (co-authors coding) were performed to identify the main domains or context in which the concept “open banking” is used and, second, a discursive analysis to determine the formal elements required for a comprehensive open banking definition

We are confident that these changes will improve the structure of the article.

**2.2. Motivation**

Despite a growing regulatory impulse towards open banking as a remedy to improve competition in the retail banking industry worldwide (Ziegler, 2021), open banking as a research object still lacks conceptualization both on theoretical and empirical levels (O’Leary et al., January 2021). Research about open banking as a theoretical construct accelerated only in 2018 (Fig. 2 in the main article); still, what is open banking and what is not is neither clear nor as simple as it may seem.

Finding an agreement on what open banking is and ascertaining principal holistic definitions in this field is essential for further research for academia and practitioners. As stated by Zeeland and Pierson in the paper that inspired this research, “*Open Banking is contingent on different perspectives: FinTech start-ups will relish another Open Banking landscape than regulators, or bank clients. Clarification can be helpful to researchers, policy makers (sic), financial institutions, regulators, consumer rights activitists (sic) and others who wish to discuss financial data-sharing and its ramifications*” (van Zeelan & Pierson, 2021).

The primary motivation for performing this research has been to set a solid background for academia to impulse research about open banking due to the limited number of theoretical studies about the concept of open banking. Moreover, an open banking definition is needed not only for academia but also to support policymakers, financial institutions and economic agents to make choices based on solid grounds. In the case of open banking, this is especially relevant, considering its economic impact. For example, the implementation cost of open banking regulations for Westpac, a large bank in Australia, was estimated (Barbaschow, 2018) between USD 105M and 140M. Yet there is no single piece of research analyzing the returns of this investment neither for the customers nor the financial system.

In the “Step-by-step personalized response to reviewers”, we provide specific answers to the comments raised, but we deemed it necessary to explain the overarching purpose of our research.

**2.3. Methodology**

Both reviewers have shown concerns about using two methodologies and how this approach results in a non-clear methodological approach. Neither the bibliometric nor the discourse analysis approach stand-alone yielded satisfactory results. However, the combination of both allowed us to identify first the 282 relevant papers after a co-reading and coding after a discussion and extract from them the key factors of the definition of open banking based on the discourse analysis.

If applied to a corpus of articles focused on analyzing the concept of open banking, the bibliometric analysis could have been sufficient. However, the literature analyzed deals with multiple perspectives of open banking beyond the definition, such as origins, implementation or implications. Thus, the outcome of the analysis is not the main domains within the definition of open banking but the different contexts and connotations of open banking.

As for the discourse analysis, our main concern with this approach stand-alone was the validity. Only 48 definitions were found in 282 articles, representing a mere 17% of the population. To ensure the validity of the results, we needed to verify a fundamental fit between the outcome of our analysis and the existing connotations found within the current literature.

Balancing the simplicity of using only one technique versus the upside of combining both approaches, we decided to follow the two-tiered process for a more accurate and valid definition.

However, as further explained in the “Step-by-step personalized response to reviewers”, we have implemented all the changes needed to better explain our method in the article and the validity.

**3. Step-by-step personalized response to reviewers**

**Response to Reviewer #1**

| **Reviewer #1 Comment** | **Response to Reviewer #1** |
| --- | --- |
| **Major concerns** | |
| **1. Motivation:** this paper needs to find additional motivations and implications. Consider, for example, why integrating the definition of open banking is essential for practitioners and scholars. | First of all, we want to thank the reviewer for the comment. The vision also entirely coincides with comments 2 and 7 of Reviewer 2.  Open banking is a complex phenomenon and, as such, analyzed by multiple academic disciplines, as shown by the distribution of articles according to academic discipline (Fig 5 in the main article). However, there is no integrative definition of open banking in the academic literature. As a matter of fact, existing definitions are idiosyncratic, fragmented and highly dependent on the academic field they were conceived.  The lack of an adequate definition is a recognized fact in the literature. O´Leary (2021) explained, "*While there are many working definitions of Open Banking in the industry, there has been little effort to understand what it actually is within academic circles*". (O'Leary et al., January 2021)  This lack of specification of the object of study has two significant negative consequences. First, it generates confusion with related terms such as open data, open innovation or banking as a service. Also, the lack of a shared definition between different academic disciplines in academic environments can result in a lack of collaboration (Smaiziene & Jucevicius, 2013).  These consequences translate into fragmented and inconsistent research: only 57 of the 282 articles analyzed have been published in ranked journals, and just 12 of these 57 articles were published in Q1 academic journals.  This situation prevents the academy from analyzing essential aspects such as the degree of adoption of services based on open banking by end customers or the change in competitive dynamics in markets where open banking has been a reality for several years.  In parallel, supervisory regulators worldwide continue to promote open banking models. The European Union is analyzing the convenience of extending the current open banking directive, PSD2, to all financial products beyond current accounts (PSD3) or even to non-banking sectors (Digital Markets Act) (Cabral et al., 2021). Reference financial systems such as the United States or Canada, which do not yet have open banking regulations, are studying the convenience of promoting them  For all of the above, we consider it critical to cover the existing gap in the literature of a holistic definition of open banking that can promote collaboration between disciplines and geographies and allow the academic literature in this area to be promoted. |
| **2. Literature review:** the literature review section is too long. It may be helpful for the authors to re-organize this part, especially to articulate the literature gap and what this paper adds to it. In other words, author may combine section 2.1 Literature review and section 2.2 research question. | Thank you very much for this comment. We agree with this observation and have re-organized and rewritten this section and combined sections 2.1. Literature Review and 2.2. Research question into 2. Literature review and research question.  This section is now focused on identifying the recent high interest in the concept of open banking and the gap in the literature on a multidisciplinary and complete definition of open banking.  This gap is relevant for developing robust academic literature and is also highly important for regulators, financial institutions, and end customers who might consider data sharing benefits. We have included the following paragraphs in the Literature review and research question section:  *“There are only three publications devoted to establishing a definition of open banking. Van Zeeland et al. (2021) follow a bibliometric and discourse analysis approach, but they fail to propose a definition, concluding that:*    *“Open Banking could be all kinds of things, from a remedy to an ecosystem, or most often: a (business) model of some sort. Its purposes are considered to be providing new (‘better’, ‘customer-centric’) services to customers and improving competition in the banking market by letting ‘third parties’ in.”*  *O’Leary et al. (2021), building on an open data lenses approach, propose the following definition:*  *An initiative which facilitates the secure sharing of account data with licensed third parties through Application Programming Interfaces (APIs), empowering customers with ownership of their own data. The initiative aims to increase competition in retail banking by developing innovative products and services which will bring increased value to customers. (O'Leary et al., 2021)*  *The proposed definition presents several flows. First of all, “initiative” is an undetermined concept, considering existing open banking regulatory frameworks worldwide. Second, it includes a specific technology (APIs) when open banking frameworks are independent of the underlying technology (e.g., PSD2 in the EU does not prescribe any particular technology to implement open banking). Finally, data ownership is a prerequisite of open banking, not a consequence. Thus, the proposed definition is incomplete and questionable.*  *Finally, Laplante and Kshetri (2021) approach the need for a definition of open banking but do not provide a generalized definition other than describing the phenomenon as:*  *“Open banking describes a special kind of financial ecosystem. The ecosystem provides third-party financial service providers open access to consumer banking, transaction, and other financial data from banks and non-bank financial institutions through the use of application programming interfaces (APIs).”*  *Thus, a generalized definition of open banking is a relevant gap in the academic literature that needs to be filled.”*  *[…]*  *“In summary, this research aims to review and systematize existing literature on open banking and analyze current open banking descriptions to induct a generally applicable definition. A particular contribution of this study is that it tackles the research question through a multidisciplinary approach, integrating views from different knowledge domains and through mixed quantitative-qualitative techniques, specifically bibliometric research and discourse analysis.”* |
| **3. The methodology section** in this paper does not use self-developed methodology, so section 3, especially section 3.1, is way too long and gives too much background information. Maybe the author could put some background information in the appendix instead.  Additionally, I am not sure what is the advantage of the two methodologies authors applied and why they fit into the research question. This part of the paper should be emphasized more than introducing the background knowledge. | We acknowledge the excessive length in the methodology. Consequently, we have moved a significant part of the former section 3.1. to a second appendix, which improves the readability of the section considerably.  Regarding the concurrent methodologies, our conceptual approach was as follows. The primary target of the article was to propose a generalized definition for open banking through the analysis of existing definitions, following a discourse analysis similar to (Fernández-Montes de Oca, Ana Isabel et al., 2021) or (Pastrana et al., 2008). After analyzing existing literature, we concluded that a significant part of the literature (232 articles out of 282) neither defined the term nor leveraged an existing definition for open banking. Limiting our contribution to a discourse analysis of the current definitions would raise the question of the validity of the outcome of our investigation for the remaining 232 articles. Thus, we structured our research into three stages. First, we applied bibliometric techniques to identify the conceptual domains related to open banking (content-driven approach). Second, we performed the discourse analysis to determine the formal elements required for a comprehensive open banking definition. Finally, we proposed a generalized definition of open banking that contained all the formal elements identified in the previous step and functional for all the conceptual domains identified during the first step. |
| **Minor concerns** | |
| **Minor concern** 1:  Page 7, line 10 “Existing literature can be classified under four different perspectives: regulatory, technological, economic, and managerial.” doesn’t need to a separate paragraph. | As part of the reorganization of the article, all the paragraphs in the literature review section have been merged into a single paragraph. |
| **Minor concern** 2:  Notions should keep consistent. Page 27, line 16 “Third, Open Banking enhances the entrance to the banking market of non-banking players…” Please make sure the notions are consistent, i.e., whether use capital letters. | We apologize for this editorial error. We have reviewed the whole document to ensure consistency. |
| **Minor concern** 3:  The section 5. Discussion and conclusions could be shortened. The current version of section 5 is too long, and repeated information is mentioned. | Thank you very much for this comment. We have rewritten this section, eliminating all repeated information. |

**Response to Reviewer #2**

| **Reviewer #2 Comment** | **Response to Reviewer #2** |
| --- | --- |
| **Comment 1:**  This paper is not very well organized and may confuse your audience. For instance, the authors mention that the existing literature can be grouped into four topics in the Introduction section. In addition, the Literature Review section also includes a review of prior literature under four different perspectives. So, I thought the prior literature groups these topics/perspectives. But when I read the Results section, it seems that these four topics are grouped based on your clustering analysis. Authors should clarify how you group prior literature into those topics. If the prior literature suggests it, what are the differences between this and the clustering results? If those are the output of the clustering analysis (your findings), they should be discussed in the Results section instead of the literature review. | First of all, many thanks to the reviewer for the comment. We fundamentally agree with it, and we are sure its implementation significantly improves the flow of the article.  Our literature review has been carried out across all academic disciplines. Considering the multidimensional nature of the open banking phenomenon, the academic literature that studies it emanates from four different domains: Regulatory, Technology, Economy, and Management. However, the number of publications by discipline is still minimal. This limitation is one of the main reasons we decided to pursue a bibliometric approach instead of a traditional one for the literature review. Figure 5 in the article shows the distribution of publications according to the scientific discipline of origin. This classification was made according to the categorization of the publications made by Scimago.  Clustering analysis has been developed regardless of the origin of the literature, as our approach only considers the distance between words and their frequency. Probably our statement, "Although cluster definitions do not exactly match knowledge domains, both spaces have a strong relation", can lead to confusion. There is no one-to-one correspondence between clusters defined and the scientific disciplines from which analyzed literature emanates.  Therefore, we have proceeded to rewrite the paragraph and relocate it to the "discussion" section to avoid inducing misinterpretation. The paragraph now reads as follows:  *“A clustering analysis to identify the conceptual domains around the open banking definition is also a valuable contribution. As an unsupervised learning methodology, clustering analysis returns an objective output, eliminating pre-classification biases. Moreover, the clustering approach unveils all the critical factors behind the open banking concept, supporting our proposal of an integrative definition valid across all disciplines and realizations of open banking. Consequently, although there are strong linkages between cluster 1 (Business model/Platform) and cluster 4 (Regulation) and the academic literature emanating from Business Management and Social Sciences-Law respectively, Cluster 2 (Data sharing) and Cluster 3 (Fintech) unveil purely transversal conceptual domains, multidisciplinary in nature that do not match with a single academic field and that could not have been identified without the clustering approach.”* |
| **Comment 2:**  The motivation of the study is underdeveloped. On page 6, there is an initial definition of open banking. So, what is the motivation behind this study to propose a generalized definition? Does the existing one have any limitations? The authors also discuss the lack of evidence about the benefit to end customers. But I don’t think this study answers this question by proposing a definition. So, I would recommend authors re-organize the literature review section and highlight the relevant motivations of this study. | To begin with, we want to thank the reviewer for the comment. The authors agree that the lack of discussion of the Open Banking Initiative leads to questions about the motivation for the article. By nature, a regulatory definition does not apply beyond the legal system it is circumscribed to. Therefore, the definition proposed by the British regulator cannot be considered in any way universal in scope. The OBI approach should be understood more as a description of the remedy than a conceptualization of the phenomenon.  As for the comment regarding the discussion of the lack of benefits for the end customer, we also agree with the observation. During our literature review, we have not found any material research regarding the benefits of open banking. Nevertheless, it is not the purpose of this paper to enter into this matter, so we have removed the reference in the introduction and the literature review.  Nevertheless, there is an insight that comes out of the investigation. Only 29.7% of the 48 definitions consider banking clients the subject of open banking. This fact is somehow concerning. Open banking relies on the willingness of the banking clients to share their banking information. Symmetrically, this right translates into an obligation for the current custodians of this data, the banks, to make this information available. As for the third parties that may access this data, open banking regulations specify the requirements to access the data. However, these third parties' right to access data emanates from client permission.  Thus, we believe this misunderstanding of the nature of open banking should be further analyzed. It is mentioned in the discussion section as an area to investigate in the future.  Additionally, we have completely reorganized the “Literature review and research question” section to clarify the motivations and contribution of this study. |
| **Comment 3:**  The sample period of the 282 documents is from 2003 to 2021. However, the concept of “open banking” was first introduced in 2015; it is possible that the term “open banking” referred to something different in the early studies? Authors may want to include an explanation to eliminate some concerns about the sample validity. | Thanks to the reviewer for the comment. We agree that the date of inception of the open banking term can create confusion.  The origins of the term open banking are uncertain. Many authors relate it to the "open" movement, especially with "open data" (O'Leary et al., January 2021) and "open innovation" (Omarini, 2018), which started at the beginning of the 21^st^ century (Chesbrough, 2003).  Our research traces the term's origins back to Simon Redfern, who created the Open Banking Project in 2012. However, in our interactions with Mr. Redfern, he confirmed that he started thinking about the term in 2003. Additionally, Jakovljevic et al. (2002) already discussed financial aggregation and open finance, so we decided to include articles from that date. (Redfern, 2021)  In any case, our research methodology consisted of the integral reading of all the articles to ensure the validity of the bibliometric analysis.  The database includes the following articles before 2015, all of them within the scope of the proposed research: (Jakovljevic et al., 2002), (Molyneux, 2002); (Eisenmann et al., 2009); (Andrieux, 2013).  To eliminate concerns about the validity, we have included the following paragraph:  *“Although the first open banking regulation was approved in 2017 in the UK (Basso et al., 2018), the concept's origins are uncertain. Simon Redfern founded the Open Bank Project in 2012 (Asli, 2012). But even before that, there have been articles in the literature containing references to “open finance” and “financial aggregation” since 2002 (Jakovljevic et al., 2002). Consequently, our database includes articles about “open banking” since 2002.”* |
| **Comment 4:**  48 definitions were extracted during the bibliometric analysis. However, it is not clear how these definitions were identified and extracted. Does the authors manual read all the abstracts or apply any algorithms? Were similar definitions extracted from different papers combined? As less than 20% of the papers have a definition, is that possible that most papers provide a definition in the main body instead of abstract? | We want to thank the reviewer for this comment because we clearly did not explain adequately in the article the approach followed.  As explained in the second part of this Rebuttal Letter, this research leverages two different approaches. Firstly, we tried to identify the main conceptual domains included in "open banking" by applying bibliometric techniques. The bibliometric engine used was VoS Viewer, which analyzed only publication abstracts. In parallel, we read the 282 articles and found 48 definitions of open banking, usually found in the main body of the articles. We analyzed these 48 definitions through discourse analysis techniques rooted in the academic literature. Thus, similar definitions were not combined but analyzed separately. All the definitions analyzed are included in the annex.  The reason to follow this approach is two-tiered. First, during our research, we realized that there was no definition of open banking referenced by many publications, and thus, a traditional approach was insufficient. Additionally, the definitions used in the different publications were idiosyncratic, partial and sometimes even contradictory. Consequently, we hypothesize that the lack of a share definition limits the development of academic literature around open banking.  To eliminate concerns about the process, we have included the following paragraph:  Section 3.1.  *“After that, entire articles are read to reject false positives, obtaining a final list of 282 documents from 2002 to 2021 (Fig. 1). The dataset is uploaded to RefWorks, a commonly used reference manager software (Basak, 2014). During this process, 48 definitions of “open banking” are identified.”*  Section 3.2.  *“During the bibliometric analysis data set-building process, 48 definitions of “open banking” are identified, each cited in just one article.”* |
| **Comment 5：**  I would recommend authors provide more details about the clustering approach and interpretation of each cluster. The main idea is to ensure validity. Do multiple authors code them independently and achieve an agreement? It seems to me that the interpretation is kind of arbitrary. For instance, the last cluster is coded as regulation. But except for the keyword “regulation”, other keywords like “payment”, “transaction”, “market” are somehow irrelevant to the topic of regulation. | As adequately pointed out by the reviewer, we did not provide enough explanation regarding the coding process.  After performing the clustering analysis, both authors independently interpreted the outcome of the clustering algorithm and discussed appropriate coding. The pre-defined criterium was that at least 50% of the terms should be unmistakably assigned to a cluster:  Cluster 1: bank; model; innovation; industry; change; platform. (BUSINESS MODEL/PLATFORM: all terms are related to new business models and a new way of banking).  Cluster 2: open banking; data; consumer; framework; system; information. (DATA SHARING: all terms are related to open access to clients and third parties to share data that previously was not possible)  Cluster 3: fintech; company; financial service; economy; banking service; implementation. (FINTECH: new players and competitors are linked to open banking using technology to make possible de innovation and sharing)  Cluster 4: psd2 regulation; market (referred to PSD2, as the Directive that aims at developing a "single European payments market"); EU (i.e., European Union); payment (referred to "Payment Initiation Services Provider" or PISP, a newly PSD2 regulated actor); account (referred to "Account Information Services Provider" or AISP, a newly PSD2 regulated actor). (REGULATION: Banking is a regulated market all over the world, and all the terms are related to legislation in the financial sector to open de access to banking data)  Although cluster coding is always subject to interpretation, both researchers' high level of coincidence in the cluster coding led to the proposed coding and experience in the Banking sector and financial institutions.  We have included an explanation of the process followed in the article as follows:  *“Before the coding, both researchers agreed on a coding method: based on heuristics, assigning to the cluster a description that explained at least 50% of the terms included in each cluster. Both researchers performed independent coding, and the results were compared and discussed to obtain the proposed interpretation.”* |
| **Comment 6:**  In the analysis of definition, this paper decomposes 48 identified definitions into 8 elements. How do those 8 elements identified? Are they developed by authors or based on literature? Authors may need to provide additional explanations. | There is no consolidated academic literature regarding how to analyze a definition. Thus, the authors followed the methodology proposed by several articles aiming at building a definition: "Deforestation" (Fernández-Montes de Oca, Ana Isabel et al., 2021), "Enterprise architecture" (Saint-Louis et al., 2019), "Palliative care" (Pastrana et al., 2008) or "Region" (Schmitt-Egner, 2002).  In all of the cited examples, the outcome of the discourse analysis is two-fold: the specific elements that structure the definition and the definition itself.  Thus, we followed a similar approach, obtaining a definition structure for open banking as a byproduct of the analysis and the definition itself as the main product of the research.  We have adjusted the article to implement the proposed comment in the following way:  *“Existing literature does not provide a framework to analyze “open banking” or similar definitions. Following similar approaches in the academic literature (Fernández-Montes de Oca, 2021; Pastrana et al., 2008), the authors proceed to build an ad-hoc framework: the “open banking integrated definition framework” based on induction from the 48 existing definitions. This process identifies eight elements in which all current definitions can be decomposed.”* |
| **Comment 7:**  The findings of the paper make potential contributions to the literature. However, authors did not systematically discuss their contributions to the literature. I would recommend including a paragraph of contribution in the Introduction section. | As suggested, we have included a paragraph explaining the contribution in the introduction. We hope that this addition helps to clarify how this article fits into the existing literature and its contribution to the development of research around open banking.  The paragraph now reads as follows:  *“Our proposed generalized definition should help increase collaboration among researchers from different academic disciplines and impulse international collaboration among researchers in different geographies to analyze the open banking phenomenon.”* |
| **Minor comment:**  The format of some citations needs to be adjusted. For instance, on page 3, change “To date, … shaping forces of the future (Ziegler, 2021) (Brackert et al., 2019).” to “To date, … shaping forces of the future (Brackert et al., 2019; Ziegler, 2021).” | Citations have been adjusted to PLOS ONE required format. |
| **Minor comment:**  Many subsections are numbered incorrectly. For example, on page 12, change “2.1.2.” to “3.1.2.”. | Subsections numbering has been corrected and adjusted to PLOS ONE format. |
| **Minor comment:**  On page 13, change “5.000” to “5,000”. | We apologize for this typographical error. We have changed “5.000” to “5,000” and reviewed the whole document to ensure there were no other errors like this. |
| **Minor comment:**  On page 15, change “10.000” to “10,000”. | We apologize for this typographical error. We have changed “10.000” to “10,000” and reviewed the whole document to ensure there were no other errors like this. |

4. **References**

Andrieux, D. (2013). *Banking in the digital age.* Sopra Banking Software.

Barbaschow, A. (2018, Oct 12). Westpac predicts Open Banking to cost AU$200m to implement. *ZDNet.* <https://www.zdnet.com/finance/westpac-predicts-open-banking-to-cost-au200m-to-implement/>

Cabral, L. M., Haucap, J., Parker, G., Petropoulus, G., Valleti, T., & Van Alstyne, M. (2021). *The EU Digital Markets Act.* Luxembourg: Publications Office of the European Union. doi: 10.2760/139337 <http://www.econis.eu/PPNSET?PPN&#61;1749382520>

Chesbrough, H. W. (2003). *Open innovation: the new imperative for creating and profiting from technology*. Harvard Business School Press.

Eisenmann, T. R., Parker, G., & Van Alstyne, M. (2009). Opening Platforms: How, When and Why? Platforms, markets and innovation, 6, 131-16. Edward Elgar Publishing. doi: 10.4337/9781849803311.00013

Fernández-Montes de Oca, Ana Isabel, Gallardo-Cruz, J. A., Ghilardi, A., Kauffer, E., Solórzano, J. V., & Sánchez-Cordero, V. (2021). An integrated framework for harmonizing definitions of deforestation. *Environmental Science &amp; Policy, 115*, 71-78. doi: 10.1016/j.envsci.2020.10.007

Jakovljevic, M., Magid, S., Caravtas, C., & Meyer, C. (2002). Financial aggregation and open finance: views from South Africa's financial portals. *South African Journal of Information Management, 4*(1), 1/6. doi: 10.4102/sajim.v4i1.150

Molyneux, P. (2002). Banking and financial services. *The European Union and National Industrial Policy* (13. pp. 259-278). Routledge.

O'Leary, K., O'Reilly, P., Papadopoulos-Filelis, C., Dehghani, M., & Nagle, T. (2021). The Sustainable Value of Open Banking: Insights from an Open Data Lens. Paper presented at the 5891-5901. 10.24251/HICSS.2021.713 <https://hdl.handle.net/10125/71333>

Omarini, A. E. (2018). Banks and Fintechs: How to Develop a Digital Open Banking Approach for the Bank’s Future. *International Business Research (Toronto), 11*(9), 23. doi: 10.5539/ibr.v11n9p23

Pastrana, T., Jünger, S., Ostgathe, C., Elsner, F., & Radbruch, L. (2008). A matter of definition – key elements identified in a discourse analysis of definitions of palliative care. *Palliative Medicine, 22*(3), 222-232. 10.1177/0269216308089803

Redfern, S. (2021, Feb 17). An idea was born. 11 Years of the Open Bank Project. https://www.openbankproject.com/11-years-of-the-open-bank-project/

Saint-Louis, P., Morency, M. C., & Lapalme, J. (2019). *Examination of explicit definitions of enterprise architecture*. International Journal of Engineering Business Management 11, 1-19. doi: 10.1177/1847979019866337

Schmitt-Egner, P. (2002). The Concept of 'Region': Theoretical and Methodological Notes on its Reconstruction. *Journal of European Integration, 24*(3), 179-200. doi. 10.1080/07036330270152196

Smaiziene, I., & Jucevicius, R. (2013). Structural Composition of a Corporate Reputation Portfolio (Sustainable Development Perspective). *International Journal of Business and Management, 8*(15), 91-101. doi: 10.5539/ijbm.v8n15p44

van Zeelan, I., & Pierson, J. (2021). *The Concept of Open Banking: From a Remedy to an Ecosystem.* Brussels, Belgium: Vrije Universiteit Brussel. <https://cris.vub.be/ws/portalfiles/portal/67938789/Policy_Brief_44_final_1.pdf>

stylefix
