# Supplementary material for: Open banking: A bibliometric analysis-driven definition
Source: PLoS One. 2022 Oct 3;17(10):e0275496. doi: 10.1371/journal.pone.0275496 (PMC9529117; doi:10.1371/journal.pone.0275496)
Supplement: Attachment — Submitted filename: PONE-D-22-03690_R1 Review report.docx [file pone.0275496.s009.docx]

**Review of PONE-D-22-03690_R1**

**“Open banking: A bibliometric analysis-driven definition”**

I appreciate the authors’ responses to my previous review comments. Overall, the revisions have improved the paper, and I continue to find the paper interesting. Although I am pleased that these efforts have addressed some of my comments, a few concerns remain unaddressed.

**Comment 1:**

My major concern is still about the contribution of the study. In section 2, the authors discussed three papers devoted to defining the open banking concept. Although the authors indicated that each has some limitations, I found that the definitions developed by O’Leary et al. (2021) and Laplante and Kshetri (2021) are very similar to the one eventually proposed by this study, which makes the incremental contribution marginal. I recommend authors provide detailed explanations and strong arguments about why/how the proposed definition fundamentally differs from the existing ones. In addition to the definition, this paper also finds some interesting observations via bibliometric analysis and clustering analysis, which could be highlighted as additional contributions.

**Comment 2:**

I recommend authors include a summary of the research method, main findings, and contributions in the introduction section.

**Comment 3:**

The literature review section is still very underdeveloped. Authors barely discussed any background of this concept or prior literature in this field but three papers devoted to defining the open banking concept to develop the motivation. As more than 200 papers identified in this field, I am curious about what all other articles are about. I think it is necessary to briefly discuss some of them and help audiences build a basic understanding of the field/literature.

**Comment 4：**

3.1.2. describes that “During this process, 48 definitions of “open banking” are identified”. However, it is confusing how those 48 definitions are identified until section 4.3, in which authors state “that final 282-document dataset was manually read, searching for formal or idiosyncratic definitions of open banking, the result of which is 48 definitions”. I recommend authors provide more explanations in section 3.

**Comment 5:**

In addition, the authors mentioned that “due to library subscription restrictions, the analysis is performed only on the titles and abstracts” (row 210), while authors said in the response letter that “… we read the 282 articles and found 48 definitions of open banking, usually found in the main body of the articles.” A clarification is needed.

**Minor comment:**

The font used is not consistent in the paper. For instance, row 99 and row 104 are different fonts.
