# Supplementary material for: Open banking: A bibliometric analysis-driven definition
Source: PLoS One. 2022 Oct 3;17(10):e0275496. doi: 10.1371/journal.pone.0275496 (PMC9529117; doi:10.1371/journal.pone.0275496)
Supplement: Attachment — Submitted filename: Response to Reviewers.docx [file pone.0275496.s010.docx]

July 18, 2022

Tawei Wang

Academic Editor

*PLOS ONE*

Dear Editor:

First, we would like to thank the referees in this second review for all the comments, suggestions, and ideas they have given us to improve our work. This paper is considerably more transparent after implementing recommended changes, and its motivation and contribution to the literature are significantly more robust.

To provide an answer to and solve the concerns raised, in this Rebuttal Letter, we use the following structure:

1. We answer the Journal Requirements regarding formatting, including all necessary changes (e.g., citation method).
2. Then we make a step-by-step personalized response to every comment raised by Reviewer #1, using a Table to pull out the comment and in a separate column detailing our response (comments in black, response in blue, additions/changes to the article in green).

We hope that our answers, modifications, and actions in the revised version of the manuscript and Rebuttal Letter have addressed the raised concerns satisfactorily.

Again, thanks for the suggestions, comments, and wishes.

Yours sincerely,

Gorka Briones de Araluze

DBA Candidate

Universidad Pontificia Comillas.
Alberto Aguilera, 23, 28015, Madrid, Spain

[gbrionesde@comillas.edu](mailto:gbrionesde@comillas.edu)

(Corresponding author)

1. **Journal Requirements**

**1.1. Please review your reference list to ensure that it is complete and correct. If you have cited papers that have been retracted, please include the rationale for doing so in the manuscript text, or remove these references and replace them with relevant current references. Any changes to the reference list should be mentioned in the rebuttal letter that accompanies your revised manuscript. If you need to cite a retracted article, indicate the article’s retracted status in the References list and also include a citation and full reference for the retraction notice.**

The reference list has been reviewed to ensure its completeness. So far, no retracted paper has been cited or removed. The references that appeared in the first version and not in this one are only due to editing changes and implementation of reviewers’ suggestions and comments.

**2. Step-by-step personalized response to reviewers**

**Response to Reviewer #1**

| **Reviewer #1 Comment** | **Response to Reviewer #1** |
| --- | --- |
| **Major concerns** | |
| 1. **Contribution**  - My major concern is still about the contribution of the study. In section 2, the authors discussed three papers devoted to defining the open banking concept. Although the authors indicated that each has some limitations, I found that the definitions developed by O’Leary et al. (2021) and Laplante and Kshetri (2021) are very similar to the one eventually proposed by this study, which makes the incremental contribution marginal. I recommend authors provide detailed explanations and strong arguments about why/how the proposed definition fundamentally differs from the existing ones. - In addition to the definition, this paper also finds some interesting observations via bibliometric analysis and clustering analysis, which could be highlighted as additional contributions. | We want to thank the reviewer for the comment. We hope that after implementing the proposed changes, the motivation for this paper is more robust and its contribution more clear.  Starting with O’Leary et al (2021), the proposed definition presents several flows. First of all, “initiative” is an undetermined concept, considering that there are existing open banking regulatory frameworks worldwide. Second, it includes a specific technology (APIs) when open banking frameworks are independent of the underlying technology (e.g., PSD2 in the EU does not prescribe any particular technology to implement open banking). Moreover, data ownership is a prerequisite of open banking, not a consequence. Finally, the authors formulate the objective of open banking to fit the object of their investigation. Actually, they affirm that “The initiative aims to increase competition in retail banking by developing innovative products and services” when actually it seeks to increase competition, lowering the barriers to entry for new competitors through access to customer data. Thus, the proposed definition is not generalizable.  Laplante and Kshetri's (2021) definition also presents relevant problems. First, it describes open banking as an “ecosystem” but does not explain what it means by an ecosystem. Secondly, it affirms that the ecosystem “provides […] open access”, when whoever provides access is the customer, owner of the data, and never open except with prior and revocable consent. Thirdly, it affirms that the data to which access can be given is "banking, transaction, and other financial data from banks and nonbank financial institutions". However, those obliged to give access to the data in the three main open banking regimes globally (UK, EU and Australia) are banking entities with transactional accounts. Third, it states that access occurs "through the use of application programming interfaces (APIs)" when the interconnection interface is not specified at the regulatory level. Finally, it does not contain any reference to the purpose of open banking, which is a problem for interpretation purposes.  To summarize this, we have introduced a paragraph that structures the problem with existing definitions:  *“The existing definitions of open banking fundamentally present three types of problems: perspective bias, discipline bias, and purpose bias. Starting with the perspective bias, open banking is a tripartite scheme (the owner of the data, the custodian, and the third party who* *accesses it). Any general definition must consider the three agents to avoid partial or incomplete analysis of the phenomenon. Regarding discipline bias, researchers tend to confuse the context in which open banking is used in their discipline with a generally applicable definition. Thus, the technical literature focuses exclusively on the technological support of the phenomenon, the regulatory literature on its legal support, and the management literature on the possible implications for the business model. However, a generalized concept of open banking must be able to encompass all its contexts of use and not just one of the meanings. Finally, the purpose bias consists of giving open banking a specific purpose other than the one for which it was formulated: to increase competition in retail banking by facilitating the entry of new competitors. Considering the combined effect of the three biases, the definitions proposed so far of open banking do not allow the construction of solid and generalizable knowledge about the phenomenon, which is a significant caveat on its development.”*  Finally, we have described why open banking is so relevant in the retail banking industry and why missing a generalized definition is critical to closing the knowledge gap.  *“One last question is why academic research on open banking is relevant. There are no global figures for the investment required to materialize open banking. According to Tink, one of the world's leading open banking service providers (32), the average open banking expenditure for a retail bank in Europe in 2020 was €83.1 Mn. So the aggregated figure for the system should be in the range of tenths of billions annually, just for Europe. Nevertheless, we have no evidence, based on scientific studies, of the intention of customers to use services based on open banking. There is no scientific evidence on how open banking can impact value creation and distribution in retail banking. No robust academic studies explain based on what and in exchange for what customers are willing to share data with third-party providers. In short, the academy has dealt with accessory elements of open banking but not with the central aspects of the phenomenon. The lack of a robust and generally shared definition of the phenomenon allowing collaboration among researchers and a holistic view of the phenomenon, is at the heart of this knowledge gap.*  *Thus, a generalized definition of open banking together with a detailed understanding of the different contexts in which the “open banking” concept is used is a relevant gap in the academic literature that needs to be filled. A particular contribution of this study is that it tackles the research question through a multidisciplinary approach, integrating views from different knowledge domains and through mixed quantitative-qualitative techniques, specifically bibliometric research and discourse analysis.”*  Finally, as recommended, we highlight in the introduction the additional contributions of the article.  *“Our research makes several contributions to the literature. First, it supposes a review of the pre-existing literature on open banking applying bibliometric techniques. Second, a generalized definition of open banking and its four meanings (Business model, Fintech, Data-sharing, regulation) are proposed. Thirdly, the 48 existing open banking definitions are systematically analyzed, and a classification is proposed for them (Institutional, Ecosystem and Client). Likewise, generated inductively, an "open banking integrated definition framework" is formulated based on eight elements that can be applied to similar definitions. Finally, the Hirschman Herfindahl Index is used innovatively within discourse analysis to measure the degree of consensus regarding a definition.”* |
| 2. **Introduction**  - I recommend authors include a summary of the research method, main findings, and contributions in the introduction section. | We want to thank the reviewer for this suggestion as it allows potential readers to anticipate the article's content better. It has been implemented as follows:  *“This article aims to understand the contexts and meanings of the term “open banking” and, based on this insight, propose a generalized definition that can be used unambiguously in the academic literature. In order to achieve the proposed objective, two methodologies are used. First, through clustering-based bibliometric analysis, 282 academic articles are analyzed to identify the areas, contexts or meanings of "open banking". Second, applying a "discourse analysis" methodology, the 48 definitions of "open banking" found in the literature are examined, and a generalized definition of the term applicable to all open banking connotations is proposed.*  *Our research makes several contributions to the literature. First, it supposes a review of the pre-existing literature on open banking applying bibliometric techniques. Second, a generalized definition of open banking and its four meanings (Business model, Fintech, Data-sharing, regulation) are proposed. Thirdly, the 48 existing open banking definitions are systematically analyzed, and a classification is proposed for them (Institutional, Ecosystem and Client). Likewise, generated inductively, an "open banking integrated definition framework" is proposed based on eight elements (Nature; Consent, Subject, Action, Object, Recipient, Process, Purpose) that can be applied to similar definitions. Finally, the Hirshman Herfindahl Index is used innovatively within discourse analysis to measure the degree of consensus regarding a definition.”* |
| 3. **Literature review**  - The literature review section is still very underdeveloped. Authors barely discussed any background of this concept or prior literature in this field but three papers devoted to defining the open banking concept to develop the motivation. As more than 200 papers identified in this field, I am curious about what all other articles are about. I think it is necessary to briefly discuss some of them and help audiences build a basic understanding of the field/literature | We want to thank the reviewer for this comment. The initial version of this article included a short literature review that was removed in response to a reviewer's comment. In response to this reviewer's comment, we have included a shorter version of this literature review as follows:  *“Existing literature can be grouped into three blocks: regulatory, technical, and managerial. The regulatory literature analyzes the legislation that supports open banking (European Union’s Second Payment Services Directive (PSD2), UK’s Open Banking Standard, Australia’s Consumer Data Right, Singapore’s Personal Data Protection Act, India (Aadhaar and Unified Payments Interface) or similar regulatory pieces being analyzed and approved on Hong Kong, Canada, Brazil (BCB Circular No. 4,015/2020) or Mexico (Ley Fintech). Existing publications either focus on a single jurisdiction (15-18) or compare different legislations (19, 20). From a technology perspective, existing literature focuses on the underlying infrastructure (21-23) as well as on the acceptance of the open banking technology from the customer’s perspective (24-26). Managerial literature analyzes the structural changes in the demand and supply of financial services in the retail banking market consequence of open banking (7, 9, 11, 27-29). Finally, other fields such as microeconomics are also starting to analyze the phenomenon (30).”* |
| 4. 3.1.2. describes that “During this process, 48 definitions of “open banking” are identified”. However, it is confusing how those 48 definitions are identified until section 4.3, in which authors state “that final 282-document dataset was manually read, searching for formal or idiosyncratic definitions of open banking, the result of which is 48 definitions”. I recommend authors provide more explanations in section 3. | We thank the reviewer for the comment.  In order to implement the suggested comment, section 3.1.2. has been re-written as follows:  *“After that, the remaining papers are fully read with a double purpose. First, on the bibliometric side, to reject false positives of the combination of the words “open” and “banking”, obtaining the final list of 282 documents from 2002 to 2021 (Fig. 2). The resulting dataset is uploaded to RefWorks, a commonly used reference manager software (26). Second, on the content analysis approach, to extract all the definitions of “open baking” included in the dataset. Forty-eight definitions of “open banking” are identified and transcribed to an excel database (Tab. 3-6, Annex 1) (42)”* |
| 5. In addition, the authors mentioned that “due to library subscription restrictions, the analysis is performed only on the titles and abstracts” (row 210), while authors said in the response letter that “… we read the 282 articles and found 48 definitions of open banking, usually found in the main body of the articles.” A clarification is needed | We appreciate the comment and acknowledge that it can lead to confusion.  Two elements must be considered: the technique's validity, the tool's limitations, and the database used.  Starting with the question of the technique's validity, co-word analysis can be applied to any combination of four elements: titles, abstracts, keywords and full text. Most bibliometric literature chooses the title and abstract combination and excludes keywords and full text. Keywords are excluded to avoid authors’ selection bias (“indexer effect”). Full-text is usually excluded for two main reasons. Firstly, applying existing algorithms requires significant preprocessing of the articles. Second, distance measurement algorithms tend to perform worse in large documents. Additionally, it is generally accepted that abstracts’ structure keeps some consistency across the literature (Zupic & Čater, 2015). Thus, we only applied the co-wording analysis to the title and abstract of the articles.  VoS Viewer does not include a preprocessing module regarding the tool and database limitations. Thus, to perform a full-text co-wording analysis, the corpus of articles must be converted into text files. This conversion has some technical limitations (e.g., tables and charts conversion) that require significant manual intervention, distorting the benefits of a bibliometric approach. In our research, we built a database with 282 articles. However, due to library subscription limitations, at least 36 (12.7%) were non-searchable files (non-searchable pdf, compilation of screenshots and Html versions of published articles). Thus, we confirmed our choice of maintaining 100% of the articles at the title-keyword-abstract level.  In order to clarify this process in the article, we have included the following change:  *“**due to limitations in obtaining full-text searchable versions of all the articles in the data set, the co-wording analysis is performed only on the titles and abstracts. This approach is consistent with existing bibliometric literature* (Zupic & Čater, 2015)*”.* |
| **Minor concerns** | |
| **Minor concern** 1:  The font used is not consistent in the paper. For instance, row 99 and row 104 are different fonts. | Thanks for the observation. The font size was inconsistent throughout the manuscript. Accordingly, it has been revised to ensure journal requirements (font size 12) are met. |

3. **References**

Basak, S. K. (2014). A comparison of researcher’s reference management software: Refworks, Mendeley, and EndNote. *Journal of Economics and Behavioral Studies, 6*(7), 2220. <http://hdl.handle.net/10321/2202>

Borgogno, O., & Colangelo, G. (2020). Consumer Inertia and Competition-Sensitive Data Governance: The Case of Open Banking. *Journal of European Consumer and Market Law, 9*(4 (2020)), 143 – 150. 10.2139/ssrn.3513514

Brodsky, L., Ip, C., & Lundberg, T. (2018). *Open banking’s next wave: Perspectives from three
fintech CEOs.* ().McKinsey & Company, Inc.

Brodsky, L., & Oakes, L. (2017). *Data sharing and open banking.* ().McKinsey & Company, Inc. <https://www.mckinsey.com/industries/financial-services/our-insights/data-sharing-and-open-banking>

He, Z., Huang, J., & Zhou, J. (2020). *Open banking: Credit Market Competition When Borrowers Own The Data.* (). New Haven, Connecticut 06520-8281: <https://cowles.yale.edu/publications/cfdp/cfdp-2262>

Leong, E. (2020). Open Banking: The Changing Nature of Regulating Banking Data - A Case Study of Australia and Singapore. *Banking & Finance Law Review, 35*(3), 443-469. <https://search.proquest.com/docview/2435720729>

O'Leary, K., O'Reilly, P., Papadopoulos-Filelis, C., Dehghani, M., & Nagle, T. (Jan 1, 2021). The Sustainable Value of Open Banking: Insights from an Open Data Lens. Paper presented at the 5891-5901. 10.24251/HICSS.2021.713 <https://hdl.handle.net/10125/71333>

Omarini, A. E. (2020). FinTech: A New Hedge for a Financial Re-intermediation. Strategy and Risk Perspectives. *Frontiers in Artificial Intelligence, 3*10.3389/frai.2020.00063

Ramdani, B., Rothwell, B., & Boukrami, E. (2020). Open Banking: The Emergence of New Digital Business Models. *International Journal of Innovation and Technology Management, 17*(5), 2050033. 10.1142/S0219877020500339

Sivathanu, B. (2019). An Empirical Study on the Intention to Use Open Banking in India. *Information Resources Management Journal (IRMJ), 32*(3), 27-47. 10.4018/IRMJ.2019070102

Tink. (2021). *Following
the money.* ().Tink. <https://resources.tink.com/hubfs/05%20Resources/Reports/2021%20reports/OBIU/Open%20banking%20investments%20and%20use%20cases%20-%20Tink%20survey%20report.pdf>

Valarini, H., & Nakano, D. (2021). An analysis of Open Banking adoption among university students in São Paulo using the Technology Readiness and Acceptance Model (TRAM). *Exacta,* 10.5585/exactaep.2021.17453

Zupic, I., & Čater, T. (2015). Bibliometric Methods in Management and Organization. *Organizational Research Methods, 18*(3), 429-472. 10.1177/1094428114562629

stylefix
